# Surgical Cases

**Published:** 1850-08

**Authors:** Frank H. Hamilton

**Affiliations:** One of the attending Surgeons to the “Buffalo Hospital of the Sisters of Charity”


ART. II.—*Surgical Cases.* Reported by FRANK H. HAMILTON, M. D., one of the attending Surgeons to the "Buffalo Hospital of the Sisters of Charity."

*Varicose Veins — Radical operation.*—Thomas Mountfield, aged 45: admitted November 17, 1849. He has ulcers on both legs, with varicose veins. The veins are most enlarged on the right leg. In 1834, one of the veins of the right leg ulcerated and bled copiously, since which time the ulcerations have been only occasionally healed. Mountfield is a bellows-maker, and he is much of the time obliged to cease labor on account of the ulcers. Habits temperate.

With horizontal posture, stimulating unguents, diet, &c., a cure of the ulcers was effected, and he was dismissed December 15.

*February* 23.—Mountfield returned for the purpose of having an operation made, the ulcers having again opened. During the five subsequent days, he was kept in bed, and subjected to a preparative regimen. On the 28th I operated on the two principal veins below the knee by *excision;* exposing the veins by an incision of an inch in length and then completely excising the portion of vein exposed. One of the veins was cartilagenous. No ligatures were used, but the wounds were closed and the bleeding arrested by a small compress over each wound, with straps and a roller moderately tight.

*March* 1.—The bandages have been rather painful during the night. Ordered to be loosened. No hemorrhage. Absolute diet. At 2, P. M., of this day had a chill. Took at 10, P. M., proto. chlor. hyd., grs. x. jalap. pulv. grs. xv.

*March* 2.—Has sympathetic fever. Leg very tender, and edges of wound unhealthy. ℞—Cataplasma ulmi Americanæ pulv., etc.

*March* 3.—Removed all dressings except the cataplasm. *Phlebitis* has commenced.

From this time forward the wounds refused to heal, phlebitis and angioleucitis extended up the leg to the middle of the thigh, followed by superficial erysipelas and then deep, diffuse phlegmon, extensive suppuration, &c., a violent irritative fever being the accompaniment. The treatment was at first soothing and cooling. When, however, the phlebitis, &c. continued to extend, it was intercepted by girdling the limb above the line of its march with successive circles of nitras argenti and tinct. iodidi. These applications arrested its upward progress. Cataplasms of ground elm were continued to the sores, and quinia, wine, &c, were administered internally. Abscesses continued to form, and a feeble irritative fever persisted in spite of tonics, good diet, &c., for many months, and the leg was not sound, and his health restored for a long time afterwards. In short, he has escaped death most narrowly, but the varices are effectually cured!

If we enquire for the cause of phlebitis, &c. in this case, it must be looked for, I think, in the constitution of the patient and in the peculiar nature of the operation, to which phlebitis is notoriously incident, rather than to any peculiarity in the mode of operation or treatment. The operation was made according to an approved method as performed by Signor Prima and others, and for which the advantage of greater safety has been claimed by its inventor. The patient was subjected to a rigid preparative treatment. The dressings of the wound were simple and light — the pressure being no greater than was necessary to restrain a hemorrhage from veins with the limb horizontal and at rest, and as soon as, in consequence of a slight swelling, this became painful, it was loosened. The chill, which occurred within twenty-six hours after the operation, indicated thus early the unfavorable issue, and the subsequent treatment cannot, therefore, be held responsible for the phlebitis. We conclude that it is only another illustration of the danger of any attempt to close varicose veins.

*Varicose veins — radical operation.*—F. W. Van Orman was admitted to the hospital as a private patient February 6, 1850, for the purpose of being operated upon for varices. Age, 23. Occupation, master of a lake vessel. Has had varicose veins, and a large, troublesome ulcer on the same leg, for several years. His habits are temperate.

Mr. V. had subjected himself, under my directions, to a moderate diet for several days before he came to the hospital. I detained him three days in the hospital under regimen before I operated.

*February* 9.—I operated in presence of the class, by *tying* three veins, each in a different mode. First, according to the original method of M Velpeau, by passing a pin under the vein, and crossing a ligature above it in the manner of the twisted suture formerly used for hare-lip. Second, according to Chaumette and others, by passing a ligature under the vein, by means of an armed needle, and then tying the ligature over a small compress upon the vein. ("Mediate or indirect ligature.") Third, by exposing the vein and then ligating it.

The ligatures were all removed within three or four days. No inflammation of consequence followed, and the patient was dismissed, cured, on the 18th of February.

*Labium leporinum; operation.*—Infant child of Mrs. Rights, aged five months. Entered hospital December 14, 1849: has double hare-lip; extends through alveoli on both sides; the central piece of the alveoli containing the rudiments of four teeth, is carried forward upon a prolongation of the septum nasi, until it is on a line with the spine of the nose. It is covered by a narrow and short piece of integument, which is continuous with the tip of the nose. I operated before the class by dissecting up the narrow slip of integument, and leaving it attached to the end of the nose, with the view of making a columna of it at some future time. *I then*

*made a partial section of the piece of septum beyond the nares, and upon which the projecting alveolus was situated, and I was now able to bend it down so as to be in place on a line with the remaining alveoli, and so as to nearly fill up the chasm in the jaw.* This part of the operation is, I believe, original with me.

The bleeding after the section of the bone, was quite free, but it was soon arrested by cold water and snow. I now abraded the edges of the chasm, in the soft parts, and brought the lips together with two interrupted sutures and with straps. On the 23d I removed the last suture, and on the 24th erysipelas commenced, which was not arrested until a week from this date. The union, however, was perfect, and was not disturbed by the inflammation.

*March* 23, 1850.—The child was again presented, and the edge of the septum having formed a very excellent columna, I had no need of the piece of integument which had been left, and therefore cut it away. The line of the jaw was perfect, with only a slight fissure on each side of the central piece.

*Labium leporinum ; operation.*—Private patient. Augustus Herbold, aged nine months: single fissure on right side of raphe.

I operated, June 13, 1850, by excising the edges of the fissure, with scissors, and closing the wound with interrupted sutures. In this case the strain on the sutures was very slight, and I did not think it necessary to employ straps.

I removed the sutures on the fourth day, and substituted straps, which latter were continued a week longer. The cure is complete.

*Fracture of os frontis; Compound. Death.* Patrick Mathews, admitted Feb. 20, 1850 ; aged 28. Fell from a scaffold 20 feet high, striking probably upon his feet, and then pitching forward upon his forehead. The malleolus internus of the right leg was broken. The os frontis was fissured over the right brow, accompanied with a considerable wound of the integument. When admitted, he was suffering under symptoms of concussion. In consultation, it was agreed that trephining was not indicated. The next day consciousness had partially returned; he was bled sixteen ounces, moderately cathartacised, and other antiphlogistic remedies were employed. On the 23d, his symptoms indicated depression, and the plan of treatment was changed. From this time until his death, which took place four weeks after, his mind wandered, and at length it became fatuitous. He had no paralysis, except of his sphincters. He was uniformly worse under the operation of a cathartic. Enormous bed sores formed; his legs and feet became œdematous, and his evacuations were involuntary. The treatment was chiefly tonic and stimulating, with occasional counter-irritation. Proto-chlor-hyd. was also given during two weeks, in small doses. No autopsy was obtained.

*Fracture of os frontis; compound. Recovery.*—Thomas Bluett, aged 22; admitted March 19, 1850. He was thrown from a wagon, his forehead striking a stone, and a wheel passing over the back of his head. The scalp was torn in both places, but chiefly over the right eye. A fracture, with very slight depression, was found under the wound over the occipital bone. This wound was closed with one suture. The fracture of the os frontis was more depressed, yet slightly: the fissures extended in several directions; he was in great pain, being conscious, and answering questions correctly. I removed with Heys' saw a small portion of bone, but was unable to introduce the levator. I then applied an instrument made like a dentist's screw for extracting stumps, by fastening it into the center of the depressed bone, and then lifting carefully, by which the bone was immediately brought to its level. Such an instrument is often useful, but it ought not to be used where much force is requisite, or where we have reasons to suppose that the inner plate is separated from the outer, lest we lift the outer plate, and leave the inner depressed. During the succeeding week, symptoms of violent cerebritis supervened, which were subdued with difficulty by three liberal bleedings, cathartics, &c. At a later period, bed sores occurred, with involuntary evacuations, for which a moderately sustaining regimen was prescribed. Copious discharges of matter took place from the wound, and one piece of bone exfoliated. The right eye was destroyed. He was dismissed in May. In July, four months after the accident, a slight discharge continues from the wound, but he is in other respects well.

*Fracture of the os frontis; compound. Recovery.*—June 5th, 1850, M—— W——, aged nine years, (private patient, in counsel with Dr. John Trowbridge,) fell about ten feet, upon the corner of a broad stair, and fractured her skull over the left brow, producing also an extensive wound in the integuments. She walked into the house alone, and described to her mother how the accident occurred. One hour after, she was nearly comatose, doubtless from effusion of blood under the cranium. The skull was broken extensively, and while a large piece was depressed to the depth of a quarter of an inch, another portion, constituting most of the superciliary ridge, was raised, and hung down over the orbit. The depressed bone was elevated, after removing several small pieces with forceps and with the elevator. The superciliary ridge could not be replaced without removing a small portion with the saw. Owing to the elasticity of the bones at this age, it was found impossible to bring all the pieces smoothly into place, yet it was predicted that in a few years, perhaps in a few months, no deformity would exist. A cooling regimen was immediately adopted. Soon after the wound was dressed, she vomited freely. Next morning she was conscious, and has from that time steadily convalesced. Scarcely any inflammation followed the injury, which we ascribe in part to the lotions of cool water which for several days were kept constantly upon the wound. July 15th, she is well, except a slight discharge from a small orifice at the seat of the original wound. The eyes are uninjured, and the slight irregularity of the forehead is even now scarcely observable.

A comparison of these three cases shows these several points of agreement, and of difference. The frontal bone was broken in each case; slightly in the first, more in the second, and by far the most in the third. The first was intemperate; we took his bottle from his pocket at the hospital; the second was temperate, but of full habit; the third was in good habit, and, it is unnecessary to say, perfectly temperate. The first was twenty-eight years old, the second twenty-two, and the third nine. The first died in a month; the second escaped death barely, and he did not leave the hospital under two months; the third walked within a fortnight, and was well in three weeks.

*Penetrating wound of the abdomen. Recovery.*—Thomas Shepard, a mulatto boy, aged 16 years, admitted June 15, 1849. Was stabbed by a sailor about eight hours before admission, with a butcher knife. Knife entered on the left side, at the anterior edge of the quadratus lumborum, about midway between the last rib and the crest of the ileum. It was dressed by Drs. Ring and Gray; has bled some, both before, and since the dressing. Lies best on his back; restless. ℞. opium gr. i, every four hours until he becomes quiet.

*June* 16.—Has taken four grains of opium; is more quiet; hæmorrhage slight. ℞. opium gr. i, every eight hours. Diet, rice water.

*June* 18.—Dressed the wound; looks well. ℞. ol. ricini f℥ss. Discontinue opium.

20*th*.—From this time the bowels were moved every day or two, and he was sustained with broths, wine, &c. On the 3d of July, we discovered a large plug of fibrin gradually pressing its way out of the wound, the mouth of which was stretched to its utmost capacity. On the 4th, we drew it out, and found it quite firm—two inches in length by three-fourths of an inch in diameter. The wound now rapidly granulated, and healed without an untoward symptom.

It is impossible to say whether the knife penetrated an intestine or not. It is not improbable that it did, as the wound was not less than four inches in depth, and its direction toward the viscera. If so, he was saved from fæcal effusion, and death, by the opium. He had, we were informed by his mother, a strong hæmorrhagic diathesis, which determined the early use of good diet, wine, &c.

*Penetrating wound of the abdomen. Death.*—August 24, 1849, Michael Rush, private patient, aged 20 years, was stabbed with a jack-knife, about four inches below the umbilicus, and to the right. The wound was on the surface, about one-quarter of an inch in length, from which a piece of omentum of the size of a hen's egg protruded. It had already been out more than an hour, and it was too completely strangulated to permit of its return with safety. I therefore applied a ligature of harness maker's silk around the mass, close to the wound, and then cut off the omentum outside of the ligature, and reduced what remained. Opium in full doses was prescribed, and complete rest enjoined. He died in twenty-four hours, from acute peritonitis. The autopsy disclosed a wound of intestine; fæcal effusion; peritoneal inflammation; serous and fibrinous effusions. This man walked some distance after the infliction of the wound, during which time we think the escape of fæcal matter into the cavity of the peritoneum occurred.

The two last cases are recorded without having full notes before me, but I believe they are, in all essential points, correct.

*Congenital Hypertrophy of a toe—Amputation, &c.*—May 18, 1849.—Jacob Argus, (private patient,) aged ten years, a native of Chictawaga, in this county, a healthy lad. His parents are Prussians and remarkably healthy. At birth, the second toe of his right foot was noticed to be longer than the other toes; and from this time it continued steadily to enlarge both in length and breadth until the present time. It is sometimes painful when he walks much upon it, but never very troublesome. At this time it measures seven inches in circumference, and projects three inches beyond the other toes.

It has the feel of firm adipose structure; its color is natural, and the skin healthy. Its great and increasing size render him anxious to get rid of it.

*May* 18.—I removed the toe by amputation, carrying the incision to near the proximal end of the metatarsal bone, and separating the bone at this point with a chain saw. My object in dividing the bone at this point, was to get beyond the diseased structure, and also to permit the adjoining metatarsal bones, which were separated an inch or more, to approach each other. The incisions upon the plantar surface were double elliptical, including a large projection of hypertrophied structure extending nearly into the center of the foot. The bleeding vessels were numerous, but only two or three required the ligature. The wound was closed by sutures and straps, and by the roller the contiguous metatarsal bones were brought nearly together without the pressure becoming painful.

On examination of the amputated member, I found that the metatarsal and phalangeal *bones* were hypertrophied in the same relative proportion with the soft parts; and the cellular texture had degenerated into a light colored, fibrous mass, holding in its cellules, whitish fat granules. The tegumentary textures were somewhat thickened and firm.

I regard it as a species of the elephantiasis Græcorum, or Barbadoes leg, although it lacks many of the features of that disease as it appears in the tropics. It is probably a more strictly local disease than the latter, and is, therefore likely to be more amenable to local treatment. The result, at least, in this instance, has shown the correctness of the practice.

The wound healed in a month, and on the 22d of June, 1850 — more than a year after the operation, I have examined the foot, and found it sound, with no disposition to reproduce the malady. I have a cast of the foot, taken before the operation, and one taken a year after, which may be seen in my museum at the Buffalo Medical College.

